# Caffeine’s influence on vertical jump height: a real-life collegiate student-athlete approach

**DOI:** 10.1080/15502783.2025.2501063

**Published:** 2025-05-04

**Authors:** Zacharias Papadakis, Vassilios Panoutsakopoulos, Antonella Schwarz, Jose Antonio

**Affiliations:** a Barry University, Human Performance Laboratory, Department of Health Promotion and Clinical Practice, Miami Shores, FL, USA; b Aristotle University of Thessaloniki, Biomechanics Laboratory, School of Physical Education and Sport Science at Thessaloniki, Thessaloniki, Greece; c Nova Southeastern University, Department of Health and Human Performance, Davie, FL, USA

**Keywords:** NCAA, ergogenic aids, sex differences, linear mixed models

## Abstract

**Background:**

Caffeine’s ergogenic potential in vertical jumping remains contested, particularly at dosages compliant with collegiate athletic regulations. The NCAA enforces a caffeine urinary threshold equivalent to ~5 mg/kg body mass, yet evidence supporting its efficacy in enhancing explosive performance among trained athletes is inconsistent. This study examined whether acute caffeine ingestion at this threshold improves squat jump (SJ) and countermovement jump (CMJ) performance in NCAA Division II student-athletes, while exploring sex-specific responses and trial-to-trial variability.

**Methods:**

Forty NCAA Division II athletes (18 females, 22 males; 21.3 ± 2.4 years) participated in a single-blind, randomized, crossover trial. Participants ingested 5 mg/kg caffeine or placebo, followed by SJ and CMJ testing on a force platform 60 minutes post-consumption. Three trials per jump type were retained to preserve intra-individual variance. Linear mixed-effects models (LMMs) assessed fixed effects of condition, sex, and trials, with random intercepts and slopes accounting for individual variability. A priori power analyses guided sample size determination, targeting sensitivity to detect small-to-medium effects (Cohen’s f ≥ 0.2).

**Results:**

Caffeine elicited trivial, non-significant differences in SJ (β =  −0.24 cm, *p* = 0.646) and CMJ (β =  −0.71 cm, *p* = 0.183) heights compared to placebo. Males outperformed females in both SJ (Δ = 9.50 cm, *p* < 0.001) and CMJ (Δ = 11.30 cm, *p* < 0.001), though no condition-by-sex interactions emerged. Trial effects were observed, with SJ improving 2.31 cm (*p* < 0.001) and CMJ 1.05 cm (*p* = 0.014) from first to second attempts, suggesting warm-up or neuromuscular potentiation. High intraclass correlation coefficients (ICC = 0.76–0.85) underscored substantial interindividual variability. Models demonstrated robust fit (conditional R^2^ = 0.86–0.92), with sufficient power to detect medium-to-large effects (f ≥ 0.25).

**Conclusions:**

A 5 mg/kg caffeine dose, compliant with NCAA thresholds, did not enhance vertical jump performance in habituated collegiate athletes, challenging prior assertions of its universal ergogenicity for ballistic tasks. While sex differences in baseline performance persisted, caffeine did not modulate these disparities, implicating neuromuscular and anthropometric factors as primary determinants. The absence of ergogenic effects aligns with NCAA safety-focused policies but highlights the need for personalized dosing strategies and research into higher doses, genetic variability, and hormonal influences. Future studies should integrate biomechanical metrics and controlled hormonal assessments to elucidate caffeine’s nuanced role in anaerobic power sports.

## 1. Introduction

Caffeine (1,3,7-trimethylxanthine) is among the most extensively researched ergogenic aids [1], favored for its potential to enhance sports performance [1–3]. Its popularity surged notably in athletic circles after being removed from the World Anti-Doping Agency’s (WADA) banned list (e.g. monitored not to exceed a concentration of 12 μg/ml in urine or 10 mg/kg of body mass) [1,4,5], especially in aerobic performance-centric sports [1,5–7]. However, its impact on anaerobic high-intensity activities, such as jumping performance, remains less explored [2,8,9] with conflicting outcomes as it appears to be ergogenic, but not in all studies [1,5,10,11].

Caffeine’s maximal physiological enhancement of all-out exercise performance is hypothesized to occur around 60 minutes post-ingestion [1–3,5,8,10,12,13] with potential mediation through peripheral and central pathways [1,5,12,14]. Among these, the most plausible mechanism relates to the blockade of adenosine receptors, the induced arousal of the central nervous system leading to enhanced motor unit recruitment and rate coding [1,5,11,13,15].

Caffeine dosages vary greatly, from 1 to 15 mg/kg of body mass [1,5,9,11,16–20], with some studies focusing on doses less than 3 mg/kg of body mass due to their perceived safety compared to higher dosages [1,5,9,10,16,21–24]. Moderate dosages ranging from 3 to 6 mg/kg of body mass are commonly acknowledged and notably recognized for their efficacy in enhancing vertical jumping performance across various studies, showcasing a dose-independent impact [1,5,10,24–35].

High doses of caffeine, ranging from 9 to 15 mg/kg of body mass, have been investigated for their ergogenic effects. However, their practicality, as exemplified by the equivalent of 11 cups of coffee or 12 mg/kg for an 80 kg individual, alongside associated side effects such as tachycardia and anxiety, may limit their viability [1,5,9–11,17,19,20]. Moreover, even studies employing lower doses, such as 6 mg/kg of body mass, have reported adverse effects, including sleep problems, tremors, abdominal discomfort, and difficulties in communication and focus [36]. Notably, regulatory bodies such as the International Olympic Committee (IOC) and the World Anti-Doping Agency (WADA), along with affiliated entities like the National Collegiate Athletic Association (NCAA), may prohibit caffeine doses exceeding moderate levels [1,25,37,38].

The NCAA persists in classifying caffeine as a prohibited substance, imposing restrictions on urinary concentrations surpassing 15 μg/ml, a threshold notably stricter than those set by the IOC and WADA for substances classified as “monitored” and not “prohibited.” Despite modifications to policies by the IOC and WADA, the NCAA upholds stringent regulations concerning caffeine consumption in collegiate athletics. This stance prompts consideration for change and harmonization with evidence-based guidelines adopted within collegiate sports [1,38–40].

In many collegiate sports, jumping is one of the most critical components associated with sprint, strength, and performance in ball games [3,8,32,41–48]. Vertical jumps, like the countermovement jump (CMJ) and squat jump (SJ), are prevalent in testing batteries for both prediction [46,49,50] and assessment of athletes’ anaerobic high-intensity power capacities for performance profiling [1,32,47,51–54]. Studies have shown that moderate caffeine consumption (i.e. around 200 to 400 mg, equivalent to 3 to 6 mg/kg of body mass) enhances anaerobic high-intensity power jumping performance [1,5,41,55–58]. However, the observed effect size of caffeine on anaerobic high-intensity power ballistic performance (e.g. jumps and throws) tends to be modest, typically ranging from 0.17 to 0.22 (2–4%) [1,5,11,55,56,59].

The literature consistently supports the acute performance-enhancing effects of caffeine consumption on anaerobic high-intensity power, including improvements in jump height, peak power, and rate of force development, with magnitudes ranging from moderate to large [41,60–63]. Studies on this topic often contend with limited sample sizes, potentially resulting in underpowered analyses that may fail to detect statistical significance at a predetermined alpha level of 0.05, thereby heightening the risk of type II errors [64]. Utilizing trained populations in testing could mitigate this risk, as athletes typically exhibit greater reliability in performance on specific tasks compared to non-athletes, thereby enhancing test-retest reliability and minimizing type II errors. This issue underscores the critical concern surrounding the limitation of statistical power in research [1,64–66]. However, despite meta-analyses affirming its benefits even in a mixed population (i.e. trained vs. untrained) [1,5,11,56], significant knowledge gaps persist hindering a precise understanding of caffeine’s optimal use in anaerobic high-intensity power ballistic-related sports [1–3,5,6,8–11,13,21,22,67–71]. The current literature lacks investigation into potential interindividual variations in strength or anaerobic power exercise concerning caffeine’s ergogenic effects. Further exploration in this area could elucidate individual responses, informing personalized strategies for optimizing athletic performance [1,5,6,72]. Although the use of caffeine is irrespective of sex [4,73], research has predominantly focused on males, leaving a significant gap in the field [73,74]. This phenomenon may be due to the effects of estrogen and/or oral contraceptive steroids on caffeine metabolism by prolonging its “ergogenic” effects on the body [5,11,73,75–78].

Thus, further research is needed to examine the ergogenic effects of caffeine consumption at the NCAA’s upper limit (5 mg/kg of body mass) during real-life training sessions on vertical jump performance, as this would deepen our understanding of caffeine’s impact on collegiate athletic performance. This study investigated whether caffeine intake enhances vertical jump performance [5,12,47,52]. Specifically, the hypothesis proposed that the maximum NCAA-approved caffeine dosage would not influence the jumping performance, including metrics such as squat jump and countermovement jump heights. In addition, to address the lack of studies in female population [8] a subgroup analysis per sex will attempt to explore potential sex differences.

## 2. Methods

### 2.1. Study design – experimental approach to the problem

The study took place at the Human Performance and Motion Analysis Center laboratories at Barry University and under controlled ambient temperature and humidity (23 ± 1 °C and 43 ± 2 %, respectively). We employed a single-blind (i.e. to eliminate expectancy effect) [79], randomized, crossover placebo-controlled design to answer the research question. All student-athletes completed two randomized experimental conditions (i.e. caffeine vs. placebo) approximately 24 hours minimum and 1 week apart. Conditions were scheduled based on student-athletes’ personal and team’s schedule/obligations (training sessions, games, travel, etc.). To control for circadian patterns, both conditions were performed at approximately the same time of the day (i.e. ~2 hours window) [80–83]. A valid semiquantitative caffeine intake questionnaire was completed to quantify participants’ 24-hour habitual caffeine intake prior to the first experimental condition [84]. Student-athletes were instructed to continue and record their normal eating habits, especially for habitual caffeine intake, for the days preceding their testing, while they were instructed not to eat 3 hours before testing or consume any caffeine or other beverage/supplement that includes caffeine, theine, taurine or another stimulant of the central nervous system [1]. Following the completion of the questionnaire, student-athletes ingested an unidentifiable gelatin capsule with either anhydrous caffeine (Bulk Supplements®) or an inert substance – microcrystalline cellulose powder (Jovvily®). The experimental conditions started after a 60-minute waiting period that involved non-strenuous work, such as writing, reading, and watching a movie. During these conditions, they executed the SJ and CMJ testing twice, once after ingesting the caffeine and once after ingesting the placebo, and related jumping performance and kinetic variables characteristics were collected.

### 2.2. Participants

Since this study involved student-athletes, both Barry’s Athletic Director and coaches were approached to grant permission to have their student-athletes approached as part of the National Collegiate Athletic Association (NCAA) compliance and other NCAA-Athletics legalities [85–87]. After that, all potential volunteers were provided with detailed information regarding the study protocol, including the schedule and nature of exercise testing. We recruited eligible active NCAA Division II student-athletes ranging from 18 to 30 years old who were engaged in sports that involve maximal-intensity ballistic tasks in training and competition (track and field, tennis, volleyball, basketball, baseball, soccer, etc.) from Barry University (Table 1). The study protocol followed the principles outlined in the Declaration of Helsinki. Thus, all student-athlete participants were required to sign an informed consent form, and the study received approval from the Ethics Committee of Barry University (#1869779–1, 02/13/2022).

**Table 1.** Descriptive statistics.

	N	Mean	SD	Minimum	Maximum
Age	40	21.32	2.41	18	30
Body Mass (Kg)	40	75.35	13.60	55.00	109.00
Stature (cm)	40	180.20	12.07	152.40	205.00
Body Mass Index (kg/m^2^)	40	23.3		23.7	25.9
Squat Jump (cm) - Placebo	40	30.93	8.12	17	57
Squat Jump (cm) - Caffeine	40	31.10	7.22	19	52
Countermovement Jump (cm) - Placebo	40	32.98	8.56	20	56
Countermovement Jump (cm) - Caffeine	40	33.68	8.21	20	55
Female	18				
Male	22				
Basketball	10				
Cross Country	2				
Soccer	14				
Tennis	3				
Volleyball	6				
Softball	5				

Initially, 54 responded to our call, but due to personal issues, only 40 completed both experimental conditions and were subsequently used for the statistical analysis. Inclusion criteria, in addition to being an active NCAA DII student-athlete, required participants to be apparently healthy (e.g. participating in team activities and injury-free for at least three months, with no lower limb injuries) and habitual caffeine users, as defined by previously established criteria [70,84]. Exclusion criteria included inability to follow given instructions (i.e. abnormal sleep, altered their regular diet, feeling unwell, consume caffeinated beverages) and perform the experimental conditions (i.e. jumps) due to any other personal or team’s related conflict/complication. Student-athletes, due to the NCAA calendar event schedule, were either in the preparation phase or in the competition phase. Therefore, specific time was allotted for training and testing during the week. Due to the variability in the periodization between sports and since we involved female sports, we decided not to control for menstrual cycle and use of oral contraceptives [5,73,75–78].

### 2.3. Procedures

#### 2.3.1. Preliminary and pre-experimental conditions

After signing the Consent Form, student-athletes completed a validated semiquantitative caffeine intake questionnaire to evaluate their habitual caffeine consumption [84]. Student-athletes were instructed to continue, record and replicate their normal eating habits, especially for habitual caffeine intake, for 24 hours preceding their testing, while they were instructed not to eat 3 hours before testing or consume any caffeine or other beverage that includes caffeine, theine, taurine, or another stimulant of the central nervous system [1]. Since the participants were active student-athletes, they were instructed to replicate the experimental conditions based on their weekly training schedule to minimize the impact of their training regimen while remaining compliant with NCAA regulations [87]. In this session, we recorded body height and mass using an electronic scale and stadiometer (Seca 703, Seca Deutschland, Hamburg, Germany) following standardized procedures [49,88,89].

#### 2.3.2. Experimental conditions

A familiarization session was not conducted, as all student-athletes regularly perform these types of jumps throughout their years of NCAA eligibility. As previously stated, testing was scheduled in alignment with the student-athletes’ and their respective teams’ training routines to minimize disruptions to their daily activities. The only exception was ensuring the replication of the 24-hour and 3-hour pre-experimental conditions to the greatest extent possible.

**Figure 1.**
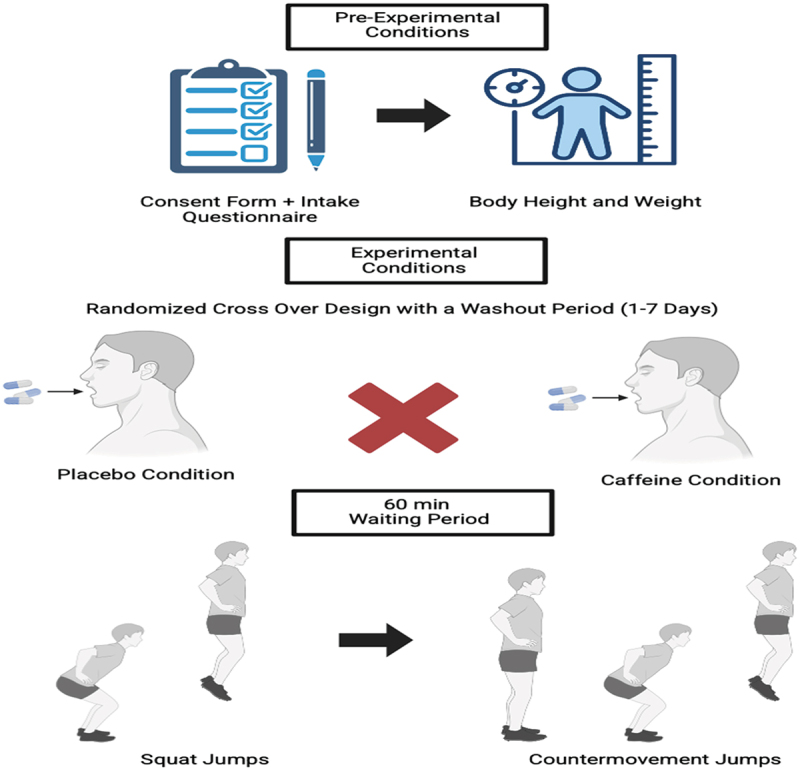
Schematic illustration of testing timeline.

A 10-minute standardized dynamic warm-up (i.e. 5 minutes of locomotor activities of jogging, running and multidirectional changes of direction and 5 minutes of dynamic stretching exercises for the upper and lower extremities) specific to jumping was performed before each condition (e.g. types of jumps). After this, 3 SJ and 3 CMJ on the force platform separated by 30 seconds of rest in-between jumps and 2 minutes between SJs and CMJs were performed. Both types of jumps were performed as previously published [41,48]. Each testing session took approximately 70–90 minutes to complete, a 60-minute waiting period after the caffeine capsule ingestion, and approximately 10–20 minutes of setup and testing (Figure 1). Although we did not formally assess the effectiveness of the blinding procedure, the research team anecdotally observed that participants’ perceptions of their assigned condition were incorrect [90].

#### 2.3.3. Caffeine

In order to strictly evaluate for caffeine effects alone and control for potential confounders related to combined ingredients (i.e. absorption rate, metabolism and overall impact [91], while controlling for standardization we used anhydrous caffeine and not a combined ingredient [1,5]. Student-athletes’ habitual caffeine consumption was controlled and evaluated based on established definition of less than 50 mg of caffeine per day in the previous months (i.e. low caffeine consumer) [70] and measured using a validated questionnaire [84]. Caffeine and placebo capsules were prepared according to standard procedures approximately 30 minutes before participants’ arrival to the laboratory containing either 5 mg•kg^−1^ of caffeine or cellulose (placebo) [1,5,11]. Capsules were identical and participants were unable to visually discern the ingredients within the capsules. The dose of 5 mg/kg was selected based on previous literature [1,5,11,41] to promote ergogenic effects while minimizing potential side effects [1,5,11,25]. Moreover, since this study employed collegiate student-athletes it had to comply with the NCAA regulations for urinary caffeine excretion of 15 μg/mL or about 500 mg of caffeine 2 to 3 hours before an event, which for reference is considered well above the World Anti-Doping Agency (WADA) (www.wada.ama.org)[37] and International Olympic Committee (IOC) [25] dosage or the amount that is deemed to be ergogenic [1,5,11,23,92]. Timing of the study was based on caffeine kinetics in the blood that reaches peak concentrations after 1 hour of intake [1,5,11,93]. Researchers verified that the capsule was consumed with 100 mL cup of water 60 minutes before testing [94].

#### 2.3.4. Vertical jump tests – data acquisition and analysis

Vertical ground reaction forces were recorded using the Noraxon AMTI® Force Plate System (myoFORCE®, Noraxon USA, Scottsdale, AZ; sampling frequency: 1 kHz). Jump types were automatically detected by Noraxon MR3 software (Noraxon USA, Scottsdale, AZ), and the data analyzed using myoFORCE Jump Analysis software (Noraxon USA, Scottsdale, AZ). The SJ and CMJ heights, derived from the body’s center of mass vertical take-off velocity, were selected for this study.

#### 2.3.5. Adverse events

This study did not employ a systematic protocol to track the incidence or severity of adverse events (AEs) during the experimental conditions. While student-athletes were encouraged to report any AEs (e.g. nausea, discomfort), a formal process for capturing and evaluating these events was not implemented [95].

#### 2.3.6. Statistical analyses

An a priori sample size determination initially relied on data from a comparable study (*N* = 25, NCAA Division I athletes) reporting Cohen’s d values of 0.38 to 0.42 for squat jump (SJ) and countermovement jump (CMJ) performance [41]. Initial sample size estimation, performed using G*Power 3.1.9.6 software (Heinrich Heine University of Dusseldorf, Dusseldorf, Germany) [96], was based on a two-tailed paired t-test [97]. With a medium effect size of *d* = 0.4, an alpha level (α) of 0.05, and desired power (1–β) of 0.80, this analysis indicated a requirement of 52 participants to detect a significant effect [8,56,68]. Recognizing the inherent complexities of repeated measures designs and to enhance experimental rigor [98,99], mitigate replication bias [100], minimize the risk of Type II errors [101], and accommodate an anticipated attrition rate of approximately 20%, we initially aimed for a minimum of 65 participants.

The study was conducted as a double-blinded, placebo-controlled, randomized crossover design, wherein each participant completed both placebo and caffeine conditions. To preserve within-condition variability and enhance sensitivity to detect small effects, all three jump trials (instead of an average or best-trial selection) were retained as separate outcome measures. This approach maintained intra-individual variance-such as potential fatigue or trial-to-trial inconsistency-rather than discarding it [102–108]. Recognizing that linear mixed-effects models (LMMs) could exploit this within-participant structure more effectively than paired t-tests, a supplementary a priori calculation was performed to approximate power requirements for a mixed-design ANOVA scenario that mimics LMM parameters [109,110].

Acknowledging G*Power’s lack of direct support for LMM power calculations, we employed a mixed-design ANOVA approximation (repeated measures, within-between interaction F-test) within G*Power. For this LMM-approximated power analysis, we posited a small-to-medium Cohen’s f effect size of 0.2 (small-to-medium) – a value deemed practically relevant within exercise performance contexts and aligning with conservative estimates from related literature reporting effect sizes ranging from 0.17 to 0.22 [32,52,55–57,106,111–114]. Utilizing the following G*Power settings: Test family: F tests; Statistical test: ANOVA: Repeated measures, within-between interaction; Effect size f = 0.2; α = 0.05; Power (1–β) = 0.80; Number of groups = 2 (male, female); Number of measurements = 6 (representing 3 trials as the repeated factor of primary interest times the 2 conditions of placebo vs caffeine); Correlation among repeated measures = 0.5 and Nonsphericity correction ε = 0.5 (conservative correction for potential sphericity violations) [115], this analysis suggested a requirement of approximately 28 participants. Adjusting this estimate to enhance rigor, address replication concerns, limit Type II error, and account for 20% potential attrition [98–101], a revised target of 35 participants was established. Sensitivity analyses were considered for the correlation among repeated measures to evaluate the robustness of the sample size estimate to variations in this assumption.

Ultimately, attrition reduced the final sample to 40 participants-below the originally envisioned 52 but above the 28–35 range gleaned from the LMM-oriented approximation. Despite not reaching 52, an LMM retains multiple advantages compared to simpler approaches. Critically, linear mixed-effects models offer superior analytical robustness compared to simpler paired t-tests and even repeated measures ANOVA. Specifically, LMMs (i) effectively utilize and model all repeated trial data within each participant, avoiding information loss through averaging or data discarding, and (ii) robustly incorporate random intercepts and slopes, thereby accommodating individual heterogeneity and enhancing the precision and minimizing bias inherent in repeated measures designs [102,103]. The inclusion of a random slope for Condition was deemed crucial a priori given existing evidence suggesting inter-individual variability in caffeine responsiveness within exercise performance contexts [116]. By leveraging this structure, the LMM approach compensates for modest sample sizes and safeguards statistical power, yielding more reliable inferences under crossover, repeated-measures designs. Consequently, the analytical advantages of the LMM approach, combined with achieved power analysis with *N* = 40, suggest that the study remained adequately powered to draw reliable inferences regarding placebo versus caffeine comparisons for vertical jump performance. However, caution in interpreting null findings, particularly for smaller effect sizes, will be warranted, acknowledging the potential for Type II error in detecting subtle effects [117].

Accordingly, two LMMs were fitted-one for SJ and one for CMJ. In both models, jump height was specified as the dependent variable, with Condition (placebo vs. caffeine), Sex (female vs. male), and Trials (1, 2, 3) as fixed effects, plus all two- and three-way interactions. To account for between-participant differences in baseline performance, a random intercept was assigned to each participant, and a random slope for Condition was included to allow for variability in caffeine responsiveness [103]. Model parameters were estimated via restricted maximum likelihood (REML), and Satterthwaite’s method was used to approximate degrees of freedom and assess significance [102]. Assumptions of normality and homoscedasticity were evaluated using Q – Q plots, residual histograms, and residual-versus-fitted plots; no transformations were made unless substantial deviations from normality were detected. Statistical significance was set at *p* < 0.05. Effect sizes (Cohen’s f) were interpreted as follows: 0.10 = small, 0.25 = medium, 0.40 = large [64,118]. All analyses were performed using jamovi (version 2.6), which implements the lme4 package in R [119–123]. This multifaceted approach balanced rigorous sample-size planning with practical constraints. By applying LMMs, researchers retained comprehensive information from each repeated trial, enhanced statistical sensitivity, and captured participant-specific patterns, thereby enabling robust conclusions regarding the influence of caffeine on jumping performance.

## 3. Results

The final sample comprised 40 NCAA Division II athletes (18 females, 22 males; age: 21.06 mean ± 2.41 SD years) across volleyball, soccer, basketball, and other sports. Attrition did not influence study’s power as our sample (*N* = 40) exceeded both the minimum (*n* = 28) and targeted (*n* = 35) required sizes for 80% power to detect small to medium effects (Cohen’s f = 0.17–0.25). Descriptive statistics are shown in Table 1.

### 3.1. Squat Jump

Forty participants completed both placebo and caffeine conditions, each with three SJ trials, yielding 240 total observations. An LMM was fitted using SJ jump height as the dependent variable, with Condition (placebo vs. caffeine), Sex (female vs. male), and Trials (1, 2, 3) as fixed effects. Random intercepts were included for each participant, along with random slopes for Condition to account for inter-individual variability in caffeine responsiveness. The final model demonstrated excellent fit with (Conditional R^2^ = 0.86, indicating both fixed and random effects; and Marginal R^2^ = 0.39, indicating variance by the fixed effects alone; AIC = 1335.98, BIC = 1391.67) converged without convergence errors or singular fits (log-likelihood = −651.99), with Cohen’s f of ~0.80 (large effect). Random intercept for participants accounted 76% of baseline variance (ICC), reflecting substantial individual differences. The main effect of Condition (placebo vs. caffeine) was nonsignificant (F_1, 38_ = 0.21, *p* = 0.646, β = − 0.24 cm, 95% CI [−1.25, 0.78], d = −0.08), indicating that the NCAA-approved caffeine dose did not significantly affect SJ jump height compared with placebo. In contrast, Sex was significant (F_1, 38_ = 31.42, *p* < 0.001, β = −9.50, 95% CI [−12.84, −6.16], d = −3.26), showing higher jump heights in males than females (9.50 cm higher). A significant main effect of Trials (F_2, 152_ = 12.86, *p* < 0.001) suggested meaningful changes in SJ jump height over the three repeated attempts (e.g. practice or fatigue effects), with Trial 1 to be 2.31 cm lower than Trial 2 (β = −2.31, 95% CI [−3.22, −1.40], *p* < 0.001, d = −0.79) with no difference between Trials 2 and 3 (β = 0.83, 95% CI [−0.08, 1.75], *p* = 0.073, d = 0.28). None of the interaction terms – Condition × Sex, Condition × Trials, Sex × Trials, or the three-way Condition × Sex × Trials – reached significance (*p* > 0.05). Residual diagnostics suggested no major violations of normality (Shapiro-Wilk’s W = 0.97, *p* < 0.001 for very slight departures) or homoscedasticity. A Q-Q plot, residual histogram, and residual-versus-fitted scatterplot revealed no severe deviations. Thus, no data transformations were performed.

**Figure 2.**
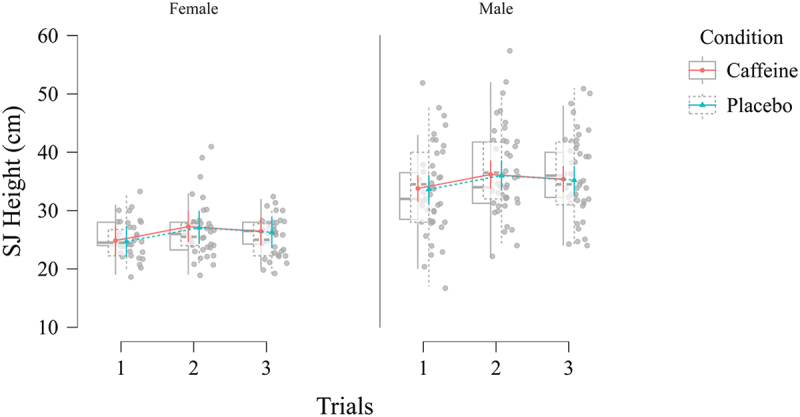
Box jitter plots for squat jump (SJ) height by sex and trial.

The maximum NCAA-approved caffeine dosage did not yield a consistent or statistically significant change in SJ jump height relative to placebo. Males exhibited higher jump heights on average than females, and performance differed across repeated trials. Men and women responded differently over the three attempts. The high ICC (intraclass correlation coefficient) indicated substantial individual variability in responses. Post hoc power analyses demonstrated sufficient sensitivity to detect medium-to-large effects (f ≥ 0.25) for primary comparisons (Table 2 and Figure 2).

**Table 2.** Squat jump height (cm) estimated means and confidence intervals.

								95% CI			
Trials		Condition		Sex		Mean		Lower		Upper	
1		Caffeine		Female		24.9		22.4		27.4	
2		Caffeine		Female		27.2		24.6		29.9	
3		Caffeine		Female		26.4		24.0		28.9	
1		Placebo		Female		24.7		22.0		27.4	
2		Placebo		Female		27.1		24.3		29.9	
3		Placebo		Female		26.3		23.6		28.9	
1		Caffeine		Male		33.8		31.5		36.1	
2		Caffeine		Male		36.2		33.8		38.6	
3		Caffeine		Male		35.4		33.2		37.6	
1		Placebo		Male		33.6		31.1		36.1	
2		Placebo		Male		36.0		33.4		38.6	
3		Placebo		Male		35.2		32.8		37.6

### 3.2. Countermovement jump

Using a similar model, 40 participants likewise performed three CMJ trials under both placebo and caffeine conditions (*n* = 240 observations). Fixed effects included Condition (placebo vs. caffeine), Sex (female vs. male), and Trials (1, 2, 3). Each participant served as a random intercept; a random slope for Condition was added to account for individual variability in response to caffeine. The final model (Conditional R^2^ = 0.92, accounting for both fixed and random effects; and Marginal R^2^ = 0.45, explained by the fixed predictors alone; AIC = 1289.50, BIC = 1345.19) also converged successfully (log-likelihood = −628.75), showing a significant random effect of participant intercept (ICC = .85, *p* < 0.001) and individual variability in the slope for Condition, with Cohen’s f of 0.90 (large effect). Nonetheless, Condition did not yield an overall main effect on CMJ jump height (F_1, 38_ = 1.84, *p* = 0.183, β = −0.71, 95% CI [−1.75, 0.32], d = −0.29). By contrast, Sex was again a strong predictor of performance (F_1, 38_ = 38.13, *p* < 0.001, 95% CI [−14.90, −7.69], d = −4.69), with males on average jumping 11.30 cm higher than females. Trials showed a significant main effect (F_2, 152_ = 4.39, *p* = 0.014), suggesting modest but notable changes across repeated attempts. Trial 1 was 1.05 cm lower than Trial 2 (β = −1.05, 95% CI [−1.81, −0.29], *p* = 0.007, d = −0.44), but no difference between Trials 2 and 3 (β = 0.15, 95% CI [−0.61, 0.90], *p* = 0.701, d = 0.06). Interaction terms – Condition × Sex, Condition × Trials, Sex × Trials, and the three-way Condition × Sex × Trials – were all non-significant (*p* > 0.05). Residual diagnostics suggested no critical violations of normality (Kolmogorov – Smirnov = 0.07, *p* = 0.173; Shapiro – Wilk’s W = 0.97, *p* < 0.001 for mild departures) or homogeneity of variance. Q – Q plots, residual histograms, and residual-versus-fitted scatterplots showed no severe deviations. Accordingly, no data transformations were performed.

**Figure 3.**
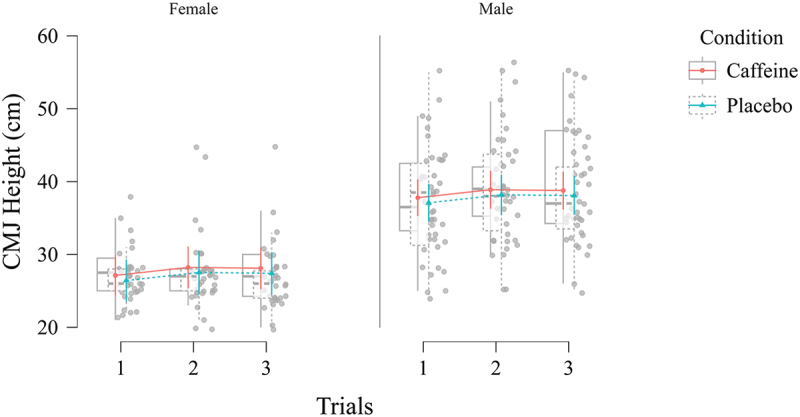
Box jitter plots for counter movement jump (CMJ) height by sex and trial.

The caffeine dose under study did not significantly alter CMJ jump height at the group level, with no consistent changes relative to placebo. Males exhibited higher jumps than females across conditions, though the condition-by-sex interaction was nonsignificant. Repeated trials produced small but significant performance changes overall. A high ICC (0.85) indicated substantial individual variability in responses. Post hoc power analyses confirmed sensitivity to detect medium-to-large effects (f ≥ 0.25) for primary comparisons (Table 3 and Figure 3).

**Table 3.** Counter movement jump height (cm) estimated means and confidence intervals.

								95% CI			
Trials		Condition		Sex		Mean		Lower		Upper	
1		Caffeine		Female		27.1		24.3		29.9	
2		Caffeine		Female		28.2		25.3		31.1	
3		Caffeine		Female		28.1		25.2		31.0	
1		Placebo		Female		26.4		23.5		29.3	
2		Placebo		Female		27.5		24.5		30.5	
3		Placebo		Female		27.4		24.5		30.3	
1		Caffeine		Male		37.8		35.3		40.3	
2		Caffeine		Male		38.9		36.3		41.5	
3		Caffeine		Male		38.8		36.2		41.4	
1		Placebo		Male		37.1		34.5		39.7	
2		Placebo		Male		38.2		35.4		40.9	
3		Placebo		Male		38.1		35.4		40.7

## 4. Discussion

This study examined whether a caffeine dose at the maximum threshold permitted by the NCAA (e.g. 5 mg/kg of body mass) would elicit an ergogenic effect on vertical jump performance in collegiate student-athletes. Contrary to some previous literature, findings revealed no significant differences in both SJ and CMJ achieved heights between the caffeine and placebo conditions.

In particular, findings suggest that the caffeine dose did not uniformly enhance SJ height, despite clear sex differences and trial repetition effects. The observed performance differences across trials could reflect a minor learning, acute neuromuscular potentiation, familiarization the jumping activity or warm-up effect, with men and women adapting differently to repeated attempts. Even though a 10 minutes standardized warm-up was implemented, these trial-to-trial improvements may indicate an insufficient warm-up protocol, where residual neuromuscular potentiation or motor learning during the early trials could have confounded treatment effects. The high ICC underscores the importance of accounting for individual variability in crossover study designs, as personalized responses may obscure group-level outcomes. This variability may reflect a complex interplay of genetic polymorphisms affecting caffeine metabolism, habitual caffeine use, training history and sporting background, and neuromuscular characteristics, each of which can modulate the ergogenic response to caffeine. Previous literature has similarly documented wide inter-individual responses in anaerobic power tasks, reinforcing the relevance of modeling individual slopes in LMM frameworks [1,2,5,8–11,64,102–106,109,110,124]. While caffeine’s lack of efficacy may reflect tolerance, dose-response thresholds, or task-specificity, the study was sufficiently powered to detect medium-to-large effects, strengthening confidence in the null findings. These results align with prior work questioning caffeine’s ergogenic benefits for explosive movements in trained populations, though further research is needed to clarify sex-specific responses to trial repetition [1–6,8–11,13,21,22,55,56,59,67–71,73–78].

In addition, our results further imply that caffeine, at the tested dose, does not reliably enhance CMJ performance, mirroring the null findings observed for SJ height. While males outperformed females overall, the absence of a significant condition-by-sex interaction suggests no sex-specific caffeine effect in this cohort. The performance changes across repeated trials may reflect a learning adaptation, warm-up benefit, or cumulative fatigue, though the small effect size limits practical interpretation. The high ICC (0.85) reinforces the critical role of individual variability in caffeine response, as seen in the SJ analysis, highlighting the need for personalized approaches in future studies. Despite caffeine’s lack of efficacy, the study’s sufficient power to detect medium-to-large effects strengthens confidence in these null outcomes. These findings align with growing evidence that caffeine’s ergogenic potential may depend on task type, habituation, or individual biology, rather than exerting universal performance benefits.

Regarding sex differences, even though males consistently outperformed females and exhibited slightly larger improvements from trial to trial, the lack of caffeine interaction effects indicates that 5 mg/kg of body mass neither improved nor impaired the observed jump height outcomes. These results align with our hypothesis that the NCAA’s upper caffeine threshold would not influence anaerobic high-intensity power tasks, suggesting that caffeine at this dosage may lack ergogenic efficacy for ballistic movements in trained athletes. Collectively these results suggest that the potential ergogenic effect of caffeine on explosive movements may be more susceptible to individual variability, optimal dosing strategies, and possible differences in the metabolic milieu of caffeine across various populations [1,5].

### 4.1. Comparison with existing literature

Our results are consistent with a body of prior literature that reports inconsistent or modest effects of caffeine on ballistic-related movements. Factors such as dosage, muscle group size, and the specific activity underscore caffeine’s complex and multifaceted nature, highlighting the imperative for further comprehensive research [11]. For both SJ and CMJ, caffeine elicited trivial but non-significant performance differences compared to placebo (SJ: Δ% = 0.55; CMJ: Δ% = 2.12), similar to what typically is reported in the literature (i.e. 2–4%) [1,8,9,11]. These observed percentage changes under caffeine align with meta-analytic findings regarding jumping performance at caffeine dosages below 3 mg/kg of body mass [22].

For instance, a study by Bloms et al. employing a similar design but with a smaller sample size (*n* = 25), found that acute caffeine consumption at 5 mg/kg of body mass augmented both SJ and CMJ jump heights in Division I collegiate athletes from sports including track and field, baseball, and football [41]. Furthermore, Donahue et al. in a single-blind crossover study involving 12 recreationally trained individuals, demonstrated that a mean caffeine dose of 5.8 mg/kg of body mass (ranging from 3.96 to 7.68 mg/kg) significantly increased both SJ and CMJ performance, with CMJ metrics being more pronouncedly affected [54]. Comparing these findings to our study, it appears a caffeine dosage threshold may exist around 6 mg/kg of body mass, approximating the upper limit of the moderately documented dosage range [1]. Conversely, a study involving ten elite volleyball players using a 5 mg/kg caffeine dose reported improved overall CMJ performance [27], contradicting our findings. Similarly, Burke et al. observed enhanced jumping performance with a 6 mg/kg caffeine dose in eleven female volleyball players [32]. While volleyball, a sport dominated by ballistic movements characterized by high motor unit firing rates and rapid force development, was represented in our sample (*n* = 6), the limited number of volleyball athletes may not have been sufficient to drive our overall results to statistical significance [1].

This is in contrast with systematic reviews suggesting negligible caffeine effects on such tasks [5,13]. Furthermore, sports heavily reliant on jumping often emphasize strength and power training to augment athletes’ jump capabilities, reflecting training specificity [125]. Masel and Maciejczyk have underscored the significance of post-activation performance enhancement in augmenting SJ performance [126]. Another factor potentially contributing to the lack of significant ergogenic caffeine effects may be the periodization strategies and seasonal timing of data collection, alongside NCAA-regulated training volumes [127,128]. Indeed, a study by Stone et al. demonstrated that five weeks of SJ and CMJ-focused training did not statistically improve jump performance in basketball and American football players [129], a finding mirrored in plyometric low-volume training protocols [130].

### 4.2. Mechanistic and population heterogeneity

Guest et al. have previously demonstrated that acute caffeine supplementation enhances various facets of exercise performance across numerous, though not all, studies. These studies often reveal small to moderate beneficial effects on jumping and other anaerobic sport-specific activities [1,92]. Ballistic movements like jumps are characterized by rapid motor unit firing rates, short contraction durations, and high rates of force development [131]. Consequently, the heterogeneity in sports backgrounds within our cohort may have influenced the observed results. Athletes from sports such as basketball and volleyball, which necessitate frequent high-intensity lower-limb actions, may respond differently to caffeine compared to athletes from sports like softball, rowing, or cross-country, which emphasize aerobic capacity and repetitive force application rather than ballistic power. This disparity arises from the inclusion of ballistic training in some sports, which has been shown to improve CMJ performance via adaptations in muscle-tendon unit kinematics and through the integration of heavy- and light-load training with stretch-shortening cycles [129,131–133]. This mechanistic variability may explain why our mixed cohort-comprising athletes from both ballistic, endurance, and mixed-focused, individual and team sports-did not exhibit uniform caffeine responsiveness. Our cohort included 5 athletes participating in individual sports (e.g. tennis and cross country), 2 athletes in endurance-based (e.g. cross country), 24 athletes in mixed-focused sports (e.g. basketball and soccer), 11 mostly ballistic-based sports (volleyball and softball), and 35 athletes participating in team sports (e.g. basketball, soccer, volleyball, softball).

### 4.3. Dosage considerations and threshold efficacy

Our null findings may be due to caffeine’s dose-dependent erogenicity. Research has demonstrated that moderate doses, ranging from 3 to 6 mg/kg of body mass, are positively correlated with enhanced jumping performance [1,6,21]. Thus, the NCAA’s 5 mg/kg threshold – while adhering to urinary regulations – may fall beneath the critical dosage needed to elicit meaningful improvements in jump performance within our specific athletic sample [38]. This underscores the practical relevance and growing acceptance of higher caffeine doses, which, while potentially more beneficial, are also associated with an increased incidence of side effects [1].

Intriguingly, Matsumura et al. found that in well-trained collegiate sprinters and jumpers, even low doses of caffeine (1 mg/kg) improved vertical jump performance in a dose-independent manner, with 1 and 3 mg/kg doses enhancing CMJ but not SJ performance, while 6 mg/kg improved both [24]. Consistent with these findings, studies in 16 basketball players [134], 18 female soccer players [135], 15 college volleyball players [45], 14 male elite Brazilian Jiu-Jitsu athletes [62], 20 experienced basketball players (10 male and 10 female) [136], and 12 male soccer players [44] have shown ergogenic effects of caffeine dosages ranging from 3 to 6 mg/kg on CMJ and/or SJ performance.

### 4.4. Sex differences and methodological nuances

An observed inconsistency within our study was the performance improvement between trial 1 and trial 2 (SJ = 1.89 cm; CMJ = 1.10 cm), indicative of potential warm-up or neuromuscular potentiation effects [137,138]. The absence of a Condition x Trial interaction may imply a lack of practice-related gains, with males exhibiting a non-significant increase across trials under caffeine, while females showed non-significant higher jumps in their second trial. This contrasts with literature suggesting caffeine enhances vertical jump performance during both single and repeated jump protocols, albeit with low to modest effect sizes [22,55]. Given the limited research on female populations, our design explicitly modeled sex and its interaction with condition and trials [8]. Subgroup analysis revealed that males outperformed females in SJ by 9.96 cm and CMJ by 11.88 cm, aligning with prior literature attributing such differences to body composition, lean mass, neuromuscular adaptations, and force production capabilities [32,47,49,139]. However, despite performance disparities, no caffeine-by-sex interaction was observed. This suggests that inherent physiological sex differences in muscle mass, power output, and neuromuscular efficiency, rather than divergent caffeine metabolism or adenosine receptor sensitivity, are the primary drivers of sex-based performance variations [1,5,73]. While research indicates caffeine may be ergogenic in jumping performance for females, particularly during the follicular phase of the menstrual cycle [8], our study did not assess or control for hormonal factors (e.g. menstrual cycle phases, oral contraceptive use). This limitation leaves open questions about estrogen’s potential modulation of caffeine’s effects, particularly in short-duration ballistic tasks [73,76–78,113]. However, this limitation also serves as a strength, reflecting real-world conditions for our sample, where team schedules are not typically structured around female athletes’ menstrual cycles.

## 5. Methodological considerations

### 5.1. Strengths

The employed crossover design minimized inter-individual variability, enhancing the study’s interpretability. Furthermore, our robust statistical methodology, accounting for intra-individual variability and the risk of Type II errors by analyzing trial effects and ensuring adequate statistical power relative to underpowered repeated-measures studies, constitutes another strength of the study. Retaining all trials within the linear mixed-effects model, as detailed in the Methods, enabled us to capture practice or fatigue effects potentially obscured by averaging [1,64,103–107,125]. This statistical approach allowed for robust inferences regarding the absence of main (condition) or interaction (sex and trials) effects. Moreover, our rigorous methodology, controlling for caffeine’s pharmacokinetic properties (i.e. testing at 60 minutes post-ingestion), mitigates pharmacokinetic misalignment as a potential confounder [1,5,75,92].

### 5.2. Limitations

This study is not without limitations. Our sample consisted of habitual caffeine users, potentially exhibiting blunted responsiveness. Despite the long-standing paradigm of caffeine’s ergogenicity [1,16,140], recent evidence suggests chronic caffeine consumption may not negate its ergogenic effects [10]. Thus, given the 5 mg/kg dosage used, it remains plausible that habitual caffeine use influenced jumping performance responses [67]. Furthermore, the within-subjects design may have inherently accounted for habituation effects [13]. Secondly, we did not control for menstrual cycle phase or oral contraceptive use, deliberately adopting a real-world approach. These factors may influence physical performance, including strength, power, and neuromuscular efficiency, due to cyclical hormonal fluctuations that impact carbohydrate and fat oxidation, and potentially caffeine metabolism, thus affecting both intra-session trial and overall jump performance [75,77]. Overall, though, the current body of evidence suggests that the menstrual cycle phase does not have a significant impact on acute strength performance or long-term adaptations to resistance exercise training in women [141]. Similarly, while oral contraceptives may influence certain muscle characteristics, they do not significantly impact responses to exercise [142]. Thirdly, the absence of formal adverse event tracking may have overlooked subtle side effects [95]. Without systematic adverse event or placebo effect documentation, we cannot preclude their presence or impact on our sample’s jumping performance, potentially affecting real-world applicability [143,144]. While placebo interventions can surprisingly enhance repeated jump performance, neglecting adverse event documentation weakens the external validity of sports science findings and introduces uncertainty [145,146]. In our study, despite employing a single-blind design, participants’ often inaccurate “guesses” about condition assignment suggest minimal expectancy effects or placebo influence (Saunders et al., 2017). Fourth, while utilizing a more sophisticated statistical analysis compared to numerous studies employing less robust methods, such as paired t-tests and repeated measures ANOVA [24,26,30,32,41,52–54,62,113,114,147–153], our sample size of 40 participants may have reduced variability in random slopes despite mitigating attrition-related power loss with the linear mixed model [154–157]. Fifth, despite rigorous protocol development, testing athletes during their regular training schedules (real-world approach with 10 minutes standardized warm-up) meant we lacked control over diurnal variations in routines, hydration, fatigue, or circadian rhythms [80,82,83], potentially dampening acute caffeine responses and its complex central nervous system interplay [1,12]. Individual training schedules, influenced by in-season or off-season status, may have introduced variability, hindering our ability to detect subtle effects. Sixth, genetic factors, like CYP1A2 and ADORA2A variants, influencing caffeine metabolism and responsiveness [1], could have affected our sample’s responses, but genetic analysis was beyond this study’s scope. Seventh, we did not assess psychological or cognitive caffeine benefits potentially impacting alertness, concentration, or perceived exertion [1,95].

### 5.3. Methodological heterogeneity in the field and analytical rigor of this study

Despite extensive research into the ergogenic properties of caffeine [1,5,73,75,92], notable discrepancies persist across studies. A significant source of this variability stems from methodological inconsistencies, including the inclusion of caffeine-naïve versus habitual coffee consumers, variations in caffeine dosages, and differing levels of control over confounding factors. Our study directly addressed several of these issues. We implemented stringent controls for habitual caffeine consumption, both in the long term (three months prior to study) and short term (24 hours and 3 hours pre-testing), carefully considering caffeine pharmacokinetics [1,5,70,84]. While recognizing that even with these controls, tolerance in habitual caffeine users remains a potential factor [1,5], our rigorous approach in minimizing pharmacokinetic variability and accounting for habituation within our within-subjects design enhances the internal validity of our findings. Furthermore, while acknowledging the potential for a dose-response relationship and that higher dosages might yield greater effects in some contexts [11,13,158]. Our choice of the NCAA-permissible 5 mg/kg dose was deliberate, aligning with real-world regulatory constraints faced by collegiate athletes, thus increasing the practical relevance and policy implications of our null findings. In contrast to numerous studies in the field employing less sophisticated statistical methods such as paired t-tests and repeated measures ANOVA, our use of a linear mixed model, retaining all trials, further enhances the analytical rigor and sensitivity of our study, allowing for robust conclusions regarding the lack of caffeine effect despite inherent trial-to-trial variability [102].

### 5.4. Trial effects and statistical approach

Another consideration is the presence of trial effects observed in our data, which may reflect warm-up, potentiation, or learning phenomena, despite participants’ routine performance of SJ and CMJ in training. For example, Guerra et al. demonstrated that post-activation potentiation on CMJ performance is enhanced by 5 mg/kg caffeine ingestion in professional soccer players [57]. Notwithstanding this intra-session variability, our analytical approach-employing a linear mixed model to retain multiple trials rather than averaging or selecting maximum jump heights-accounted for trial-to-trial fluctuations, enhancing analytical sensitivity and reinforcing the robustness of our findings regarding caffeine’s null effect [102].

### 5.5. Future research directions

Future research should explore acute caffeinated responses in caffeine-abstinent athletes, incorporate sex-specific hormonal monitoring, and investigate higher permissible dosages (e.g. 6–9 mg/kg) to clarify performance enhancement. Furthermore, incorporating biomechanical analyses – examining movement characteristics, impulse, force, velocity, power, and time metrics could delineate caffeine’s neuromuscular mechanisms beyond simple jump height measurements [159].

## 6. Conclusion

This study’s findings challenge assumptions about caffeine’s ergogenic potential in anaerobic tasks, prompting a critical reexamination of current regulatory thresholds [1,5,13]. The NCAA’s 5 mg/kg caffeine limit, while undoubtedly prioritizing athlete safety, appears to operate in a performance-efficacy no-man’s land for vertical jump performance in habituated, trained athletes, as evidenced by our null findings and converging evidence in the literature [1,5,8,9,11,73]. Our findings expose a critical tension: are we regulating a dosage that is simultaneously restrictive and functionally inert for enhancing ballistic power in this population? Given the absence of measurable performance gains at this legally sanctioned dose in our study, and supported by systematic reviews [1,5,13], the continued emphasis on a 5 mg/kg caffeine threshold for anaerobic tasks like vertical jumping warrants serious reevaluation, if not outright dismissal. Coaches and athletes diligently seeking that marginal gain might be chasing a phantom with caffeine at this dosage [1,21,22], potentially diverting resources from genuinely effective nutritional or training strategies [125,129]. Moreover, the persistent focus on a potentially inconsequential caffeine limit risks overshadowing the far more pertinent issue of substantial inter-individual variability in caffeine metabolism [1,5,16]. This variability, which genuinely dictates individual responses and the potential for adverse effects [1,73,95], demands far greater attention and personalized strategies than a one-size-fits-all, and potentially ineffective, regulatory cap. These findings compel a bold reconsideration of policy harmonization across athletic governing bodies, urging a shift from simplistic, potentially symbolic restrictions toward a more nuanced, evidence-based approach that prioritizes genuine athlete well-being and acknowledges the complex, individualized nature of caffeine response [1,11,73]. The continued pursuit of research into this inter-individual variability is not just academically valuable, but fundamentally critical for rational and effective sports nutrition policy in the 21st century [16,72,100,144].

## Data Availability

The raw data supporting the conclusions of this article will be made available by the authors on request.
